# Carbolic Acid as an Antiseptic

**Published:** 1893-05

**Authors:** 


### Carbolic Acid as an Antiseptic.

The swing of the pendulum is bringing antiseptic surgeons back to the point from which they set out. With the knowledge that wound-complications were almost invariably dependent upon contamination by living matter, efforts were directed to the finding of a body capable of preventing such contamination, without at the same time causing injury. Of all the antiseptics proposed or used, carbolic acid and mercuric chloride are the two that have shown themselves most deserving of confidence, and the second has been much more largely used than the first. Recent developments have, however, tended to demonstrate that mercuric chloride is not the perfect antiseptic that it has measurably enjoyed the distinction of being; and now comes the interesting announcement that Sir Joseph Lister (*British Medical Journal*, No. 1674, p. 163,) has practically repudiated mercurial solutions for antiseptic purposes in surgical procedures, and has returned to the use of carbolic acid, which, in the strength (1 : 20) in which it is ordinarily employed, he shows to be germicidal to most of the noxious organisms.—*Med. News.*

